# Long-term outcomes and predictive factors of achieving low disease activity status in childhood systemic lupus erythematosus: a Chinese bicentric retrospective registered study

**DOI:** 10.3389/fimmu.2024.1369969

**Published:** 2024-06-10

**Authors:** Xi Xi Yu, Jia Deng, Qiu Xia Chen, Shi Yuan Qiu, Chao Hui Jiang, Yi Qian Wu, Qin Yang, Gao Fu Zhang, Hai Ping Yang, Fei Zhao, Qiu Li, Ai Hua Zhang, Mo Wang

**Affiliations:** ^1^ Department of Nephrology, Ministry of Education Key Laboratory of Child Development and Disorders, National Clinical Research Center for Child Health and Disorders; China International Science and Technology Cooperation Base of Child Development and Critical Disorders, Children’s Hospital of Chongqing Medical University, Chongqing, China; ^2^ Department of Pediatric, Chongqing Yubei Maternal and Child Health Hospital, Chongqing, China; ^3^ Department of Nephrology, Children’s Hospital of Nanjing Medical University, Nanjing, China

**Keywords:** systemic lupus erythematosus, treat-to-target strategy, low disease activity state, predictive factors, children

## Abstract

**Background:**

This study aims to explore the clinical value of low disease activity state (LDAS) in the treat-to-target strategy of pediatric systemic lupus erythematosus (pSLE) and find the risk factors for never reaching LDAS.

**Methods:**

A total of 272 children with SLE who were diagnosed and followed up in two tertiary hospitals in China during the period from January 2012 to December 2019 were involved in this study, and the clinical presentation, pathology, and treatment were retrospectively studied.

**Results:**

The male-to-female ratio was 1:5.2, the age at diagnosis was 11.1 years (IQR, 9.8–13.1 years), the disease duration was 1.0 month (IQR, 0.5–2.0 months), and follow-up was 36.5 months (IQR, 25.7–50.9 months). During follow-up, 230 children achieved LDAS, and 42 were never been in. Male (*P* = 0.018), mucosal ulcer (*P* = 0.048), liver function damage (*P* = 0.026), cardiac effusion (*P* = 0.034), anemia (*P* = 0.048), urine red blood cells (*P* = 0.017), urinary leukocytes (*P* = 0.032), and endothelial cell proliferation in renal biopsy (*P* = 0.004)—these indexes have statistical differences between the two groups in the baseline. At baseline, endothelial cell proliferation (*P* = 0.02) is an independent risk factor for never achieving LDAS by multivariate logistic analysis. During follow-up, non-compliance was a risk factor for never achieving LDAS by comparing between groups. Children with biologics achieved LDAS at a higher rate than children without biologics (*P* = 0.038). The proportion of organ damage in patients never been in LDAS was significantly higher than that in patients who achieved LDAS (*P* < 0.001).

**Conclusion:**

Endothelial cell proliferation in renal biopsy and non-compliance during follow-up were independent risk factors for never achieving LDAS. At the end of the follow-up, the organ damage in the remission group was similar to that in the LDAS group, indicating that LDAS can be used as a target for pSLE treatment.

## 1 Introduction

Systemic lupus erythematosus (SLE) is an autoimmune disease with complex presentation, course, and prognosis (1). The disease manifestations of pediatric SLE (pSLE) are often more serious, especially in the renal, nervous, and circulatory systems (2, 3). Remission and relapse occur repeatedly in the progress of treatment.

Remission is the best target during the treatment of pSLE. However, although some patients have made the best effort to receive long-term treatment, they are unable to achieve remission at all. On the other hand, the toxicity related to the long-term usage of corticosteroids and immunosuppressive agents brings patients serious organ damage and depression, thus reducing the health-related quality of life (HRQoL). Preliminarily, the treat-to-target (T2T) strategy, as a process of therapy to achieve and maintain a predefined treatment goal or disease status combined with clinical states and laboratory markers, has been recommended in chronic disease (4–6). In autoimmune diseases, the T2T strategy was first proposed in rheumatoid arthritis (RA) (7–9). To control disease activity, reduce relapse, decline mortality, and improve HRQoL, T2T has been proposed in the treatment of SLE since 2014 by the multidisciplinary international task force which defined various targets of SLE, including complete remission off therapy, complete remission on therapy, clinical remission off therapy, clinical remission on therapy, and low disease activity state (LDAS) (10–12).

Our preliminary study showed that 65.7% of 220 lupus nephritis (LN) children did not achieve any level of remission, which suggested that LDAS could be an acceptable alternative when remission is not achievable (13). Simultaneously, it happens that the application of reaching LDAS in the treatment of pSLE has been carried out in a few studies. A UK study (14) first demonstrated that the attainment of LDAS could reduce the risk of severe flare and new damage in childhood lupus. Another study from Thailand (15) reported the outcomes of achieving LDAS within 12 months after treatment. On the other hand, the definition of childhood lupus low disease activity state (cLLDAS) has been timely formulated by the PReS-endorsed international childhood lupus T2T task force, and the target steroid dose was adjusted to 0.15 mg/kg/day (maximum of 7.5 mg) due to the large weight difference among childhood lupus (16). However, the long-term effects of LDAS in the treatment of pSLE have rarely been studied.

We conducted a retrospective bicentric study by analyzing their clinical manifestations, therapeutic regimen, and disease states to explore the risk factors of never achieving LDAS and evaluate the long-term effects of the LDAS concept in the treatment of pediatric SLE.

## 2 Patients and methods

### 2.1 Patients

In this study, data were included on patients who visited in two tertiary hospitals from January 2012 to December 2019 and met the following criteria: age <18 years old, the Lupus Research International Clinical Collaboration (SLICC) 2012 or 2019 European League Against Rheumatism/American College (EULAR/ACR) of Rheumatology classification criteria for systemic lupus erythematosus (17), and at least 1 year of follow-up and two visits per year, no more than 6 months apart in the follow-up period from diagnosis to December 2020. The exclusion criteria were death caused by reasons except SLE or follow-up time <1 year or more than 6 months apart. We collected data on 272 pediatric patients who were diagnosed with SLE and followed up in two tertiary hospitals. The study started the clinical data collection after passing the ethical review. Ethics approval was obtained from the ethics committee of the two tertiary hospitals [project number: 2017(1284)]. A standard treatment protocol appropriate to the patient’s situation was followed during treatment (18). The detailed treatment protocol will be described in the **Supplementary Material**.

### 2.2 Clinical data collection

Clinical data of children with SLE were collected retrospectively by bicentric researchers with a Case Reported Form (CRF) designed by Center One, including main clinical manifestations, laboratory results, renal biopsy, etc. The researchers of Center One and Center Two summarized and gathered the statistics of all clinical data.

The estimated glomerular filtration rate (eGFR) was calculated using the Schwartz formula (19). SLE activity was scored by the Systemic Lupus Erythematosus Disease Activity Index 2000 (SLEDAI-2K) (20), and organ damage was assessed by the pediatric version of Systemic Lupus International Collaborating Clinics/American College of Rheumatology Damage Index (pSDI) (21). The renal biopsy report was read again by our center, and LN is pathologically classified according to the International Society of Nephrology/Renal Pathology Society (ISN/RPS) classification (2003) (22). The activity index (AI) and chronic index (CI) of each patient were scored per the semi-quantitative scoring method of the National Institutes of Health (NIH) (23).

Clinical presentation, laboratory tests, and renal pathology data at baseline level were collected as well as SLEDAI to evaluate disease activity and pSDI to evaluate organ damage. The children were followed up again at the follow-up endpoint for SLEDAI and pSDI levels.

### 2.3 Definition

The definitions of the main clinical manifestations, liver function damage, lupus pulmonary injury (24), pleural or pleural effusion, cardiac effusion, and treatment non-compliance are shown in **Supplementary Table S1**. The definitions of the outcomes of SLE are shown in **Supplementary Table S2**. Disease activity was assessed by the SLE Disease Activity Index 2000 (SLEDAI-2K), and organ damage was assessed by the pediatric version of Systemic Lupus International Collaborating Clinics/American College of Rheumatology Damage Index (pSDI).

### 2.4 Study strategy

**Figure 1:**
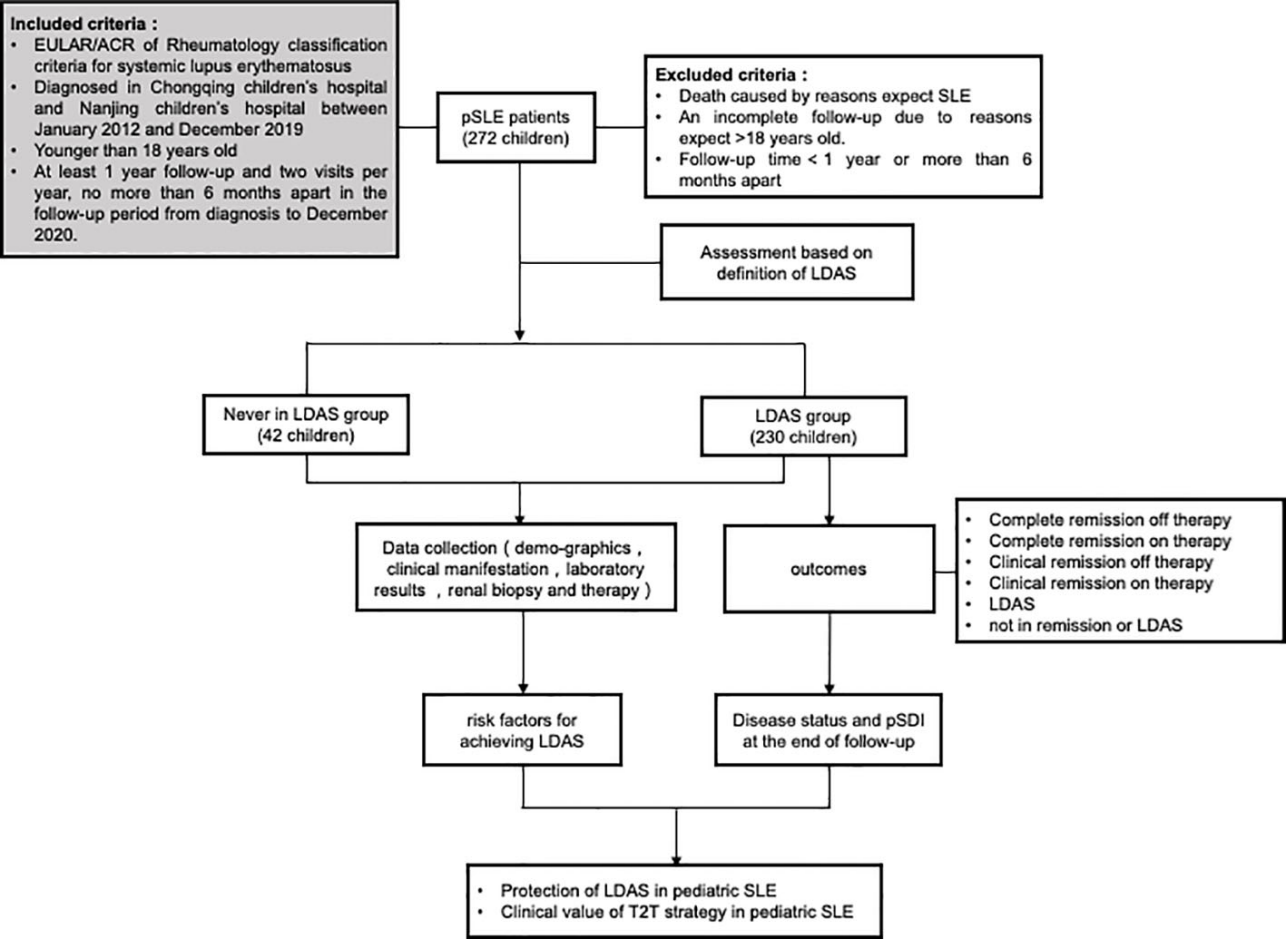
Flow of the research. Patients will be evaluated for the first time after 3 months of standardized treatment and will be followed up during each outpatient or inpatient visit. The patients will be divided into two observation groups, the LDAS group and the never LDAS group, according to their initial attainment of low disease activity status (LDAS), which lasts for more than 3 months. The last assessment was performed at the endpoint of follow-up or at 18 years of age.

Regular follow-ups were done through outpatient follow-up, inpatient treatment, and telephone follow-up. The endpoint events were (1) reaching the end time of follow-up, (2) death due to SLE or/and its associated complications, and (3) age >18 years old. Clinical data on follow-up endpoints were collected to assess whether LDAS was reached. The children were divided into two groups: those who achieved LDAS and those who never achieved LDAS. The clinical features, pathological manifestations, and long-term organ damage were compared between the two groups to investigate the risk factors affecting the children’s achievement of LDAS status and whether LDAS could replace clinical remission as a new target outcome. The flow of the research is shown in **Figure 1**.

### 2.5 Statistical analysis

All data were analyzed by using SPSS software version 26.0 (IBM Corp., Armonk, NY, USA). Continuous variables were presented as mean with standard deviation (mean ± SD) or median with interquartile range (IQR). Categorical variables were presented as frequencies and percentages [*n* (%)]. We used Student’s *t*-test and the Mann–Whitney *U*-test to compare continuous variables. Categorical variables were analyzed using chi-square or Fisher’s exact test. Univariate and multivariate analyses of risk factors were performed using logistic regression analysis. Comparisons were performed between groups using log-rank test. Data were examined in the 95% confidence level, and a *P*-value <0.05 was considered statistically significant. Figures were created with GraphPad Prism 9.

## 3 Results

### 3.1 Clinical characteristics of children with SLE at baseline

Of the 272 children with SLE, with a median (IQR) age of 11.0 (9.7–13.0) years, 228 were female (83.2%), disease duration was 1 month (IQR, 0.5–2.0 months), which means the time between the onset of clinical symptoms and the confirmation of the diagnosis by laboratory tests and renal pathological findings, and follow-up period was 36.5 (25.7–50.9) months (**Table 1**). The baseline SLEDAI was 14.0 (9.0–20.0). Follow-up time was 36.5 (IQR, 25.7–50.9) months.

**Table 1:** Demographic features, clinical manifestation, and laboratory characteristics of patients who achieved or never achieved LDAS at baseline.

	Overall (*n* = 272)	Patients who achieved LDAS (*n* = 230)	Patients who never achieved LDAS (*n* = 42)	*P*
Demographic features				
Number of patients (%)	272	230 (84.6)	42 (15.4)	
Female, *n* (%)	228 (83.8)	198 (86.1)	30 (71.4)	0.018
Age at baseline, years	11.1 [9.8, 13.1]	11.0 [9.4, 13.1]	11.4 [10.0, 13.0]	0.400
Duration at baseline, months	1.0 [0.5, 2.0]	1.0 [0.5, 2.0]	1.0 [0.5, 2.3]	0.698
Duration of follow-up, months	36.5 [25.7, 50.9]	36.8 [27.0, 52.9]	30.7 [20.4, 49.4]	0.040
Clinical manifestation				
Fever, *n* (%)	148 (54.4)	128 (55.7)	20 (47.6)	0.336
Hypertension, *n* (%)	52 (19.1)	48 (20.9)	4 (9.5)	0.086
Rash, *n* (%)	184 (67.7)	158 (68.7)	26 (61.9)	0.387
Mucosal ulcer, *n* (%)	57 (21.0)	53 (23.0)	4 (9.5)	0.048
Alopecic, *n* (%)	26 (9.6)	25 (10.9)	1 (2.4)	0.151
Hematologic involvement, *n* (%)	225 (82.7)	196 (85.2)	32 (76.2)	0.110
Anemia, *n* (%)	205 (75.4)	171 (74.4)	34 (81.0)	0.361
Leucopenia, *n* (%)	128 (40.1)	107 (46.5)	21 (50.0)	0.678
Thrombocytopenia, *n* (%)	75 (27.6)	64 (27.8)	11 (26.2)	0.827
Headache, *n* (%)	15 (5.5)	12 (5.2)	3 (7.1)	0.893
Epilepsy, *n* (%)	4 (1.5)	3 (1.3)	1 (2.4)	0.491
Mental disorder, *n* (%)	7 (2.6)	6 (2.6)	1 (2.4)	1.000
Lupus renal injury, *n* (%)	209 (76.8)	179 (77.8)	30 (71.4)	0.366
Proteinuria, *n* (%)	138 (50.7)	121 (52.6)	17 (40.5)	0.148
Hematuria, *n* (%)	119 (43.8)	105 (45.7)	14 (33.3)	0.139
Pleurisy, *n* (%)	60 (22.1)	52 (22.6)	8 (19.1)	0.609
Renal function damage, *n* (%)	49 (18.0)	40 (17.4)	9 (22.0)	0.485
Hypoalbuminemia, *n* (%)	83 (30.5)	74 (32.2)	9 (21.4)	0.191
Liver function damage, *n* (%)	105 (38.6)	94 (50.8)	11 (26.2)	0.026
Lupus pulmonary injury, *n* (%)	107 (30.3)	90 (39.1)	17 (40.5)	0.870
Pl, *n* (%)	92 (33.8)	77 (33.5)	15 (35.7)	0.778
Cardiac effusion, *n* (%)	52 (19.1)	38 (16.5)	14 (33.3)	0.034
Arthritis, *n* (%)	97 (35.7)	81 (35.2)	16 (38.1)	0.720
SLEDAI-2K	14.00 [9.0, 20.0]	14.00 [9.0, 20.3]	12.50 [7.8, 20.0]	0.324
pSDI >0	86 (31.6)	73 (31.7)	13 (31.0)	0.839
Laboratory characteristics				
WBC, × 10^9^/L	4.14 [3.0, 6.0]	4.14 [3.0, 6.1]	4.14 [3.3, 5.8]	0.923
PLT, × 10^9^/L	150.00 [98.0, 232.8]	152.50 [98.0, 231.3]	146 [89.3, 243.5]	0.933
Hb, g/L	96.4 ± 20.7	97.5 ± 20.6	90.6 ± 21.0	0.048
ESR, mm/h	41.0 [21.5, 71.0]	40.0 [23.0, 72.0]	47.5 [19.3, 69.8]	0.964
Urine RBC,/μL	13.0 [1.9, 121.0]	15.0 [2.0, 132.0]	4.0 [0.0, 62.5]	0.017
Urine WBC,/μL	10.1 [3.0, 28.8]	11.0 [4.0, 30.1]	5.0 [0.8, 28.0]	0.032
Proteinuria, g/day	1.7 [1.0, 3.0]	1.7 [1.0, 2.1]	2.3 [0.9, 4.9]	0.294
Alb, g/L	33.8 ± 8.0	33.8 ± 8.1	33.5 ± 7.5	0.733
ALT, U/L	31.0 [16.8, 56.2]	31.4 [17.0, 57.5]	29.1 [15.2, 49.3]	0.659
AST, U/L	35.2 [24.2, 63.0]	34.8 [24.1, 64.1]	37.4 [24.4, 55.2]	0.861
BUN, mmol/L	4.9 [3.6, 8.2]	4.9 [3.7, 8.1]	5.0 [3.4, 10.4]	0.967
SCr, mmol/L	46.0 [36.3, 68.8]	47.0 [36.8, 69.2]	42.0 [34.5, 68.4]	0.528
eGFR, ml/min/1.73 m^2^	152.3 [104.0, 189.7]	148.0 [103.8, 186.0]	167.5 [108.1, 214.6]	0.239
Coombs test positivity, *n* (%)	207 (76.1)	174 (75.7)	33 (78.6)	0.683
Low C3 level, *n* (%)	239 (87.9)	202 (92.2)	37 (90.2)	0.667
Low C4 level, *n* (%)	224 (82.4)	189 (82.2)	41 (97.6)	0.856

WBC, white blood cells; BUN, blood urea nitrogen; SCr, serum creatinine; eGFR, estimated glomerular filtration rate; 24hUP, 24-h urinary protein; ALT, alanine aminotransferase; AST, aspartate aminotransferase; ESR, erythrocyte sedimentation rate; C3, complement 3; C4, complement 4.

At baseline, the incidence of hematologic involvement was the highest (82.7%). Coomb’s test was positive in 207 patients. A total of 209 children (76.8%) had renal involvement. Among those, proteinuria was the most common manifestation (50.7%). Kidney function was injured in 49 (18.0%) at diagnosis, and the media eGFR of these patients was 53.35 (27.4–72.2) ml/min/1.73 m^2^. In addition, Rash (67.7%) and hyper-ESR (59.9%) were also common at baseline. Involvement in the nerve system was rarely observed, including headache (5.5%), mental disorder (2.6%), and epilepsy (1.5%). Low C3 (87.9%) and C4 (82.4%) were detected in nearly all patients. Autoantibody can be detected, including ANA (89.3%), anti-double-stranded DNA (anti‐dsDNA) antibodies (77.2%), anti-nucleosome antibodies (63.6%), anti-single-stranded DNA (anti‐ssDNA) antibodies (54.0%), etc. (**Supplementary Table S4**).

### 3.2 Risk factors of never achieving LDAS

During the follow-up sessions, 230 patients (84.6%) achieved LDAS at least once and 42 patients (15.4%) never achieved LDAS. A total of 71 cases (30.9%) achieved LDAS within 1 year, 116 cases (50.4%) achieved LDAS in 1 to 2 years, and 43 cases (18.7%) spent over 2 years to achieve LDAS. The median time to achieve the first LDAS was 15.7 (11.7–21.9) months.

The clinical characteristics of patients who achieved or never achieved LDAS at baseline are shown in **Table 1**. We found some differences between the two groups at baseline. The proportion of male patients who achieved LDAS was significantly lower than those who never achieved LDAS (*P* = 0.018), suggesting that male SLE patients have more difficulty in achieving LDAS. Because male was observed to be a risk factor for never achieving LDAS, we compared the baseline data of male and female children separately at the same time and found that female children had more cardiac effusion (**Supplementary Table S5**).

At baseline, significant differences were found in male patients, mucosal ulcer, liver function damage, cardiac effusion, anemia, urine RBC, and urine WBC between the group of SLE children achieving LDAS and the group never achieving LDAS. We also compared disease activity at baseline levels between the two groups of children (**Supplementary Table 3**) and found no statistically significant differences.

In terms of renal pathology, we further compared the pathological changes in those who achieved LDAS and those who never achieved LDAS (**Table 2**; **Supplementary Figure S1**). We found that glomerular endothelial cell proliferation under light microscopy was more apparent in patients who never achieved LDAS and was statistically different from those who did (*P* = 0.004).

**Table 2:** Renal pathological characteristics at baseline in the LDAS group and never LDAS group.

	LDAS group (*n* = 71)	Never LDAS group (*n* = 12)	*P*
Glomerular			
Mesangial cells and stroma proliferation, *n* (%)	67 (94.4)	12 (100.0)	0.399
Focal proliferation, *n* (%)	20 (28.2)	6 (50.0)	0.132
Diffuse proliferation, *n* (%)	47 (66.2)	6 (50.0)	0.280
Endothelial cell proliferation, *n* (%)	57 (80.3)	5 (41.7)	0.004
Spikes projecting, *n* (%)	11 (15.5)	4 (33.3)	0.137
Basement membrane thickening, *n* (%)	37 (52.1)	7 (58.3)	0.690
Double track sign, *n* (%)	21 (29.6)	6 (50.0)	0.163
Renal tubule			
Dilatation, *n* (%)	16 (22.5)	3 (25.0)	0.851
Atrophy, *n* (%)	11 (15.5)	3 (25.0)	0.416
Crescent	0.1 [0.1, 0.3]	0.3 [0.2, 0.6]	0.094
Renal interstitium			
Fibrosis, *n* (%)	11 (15.5)	3 (25.0)	0.416
Edema, *n* (%)	9 (12.7)	1 (8.3)	0.669
Inflammatory infiltration, *n* (%)	36 (50.7)	7 (58.3)	0.625
Renovascular			
Angionecrosis, *n* (%)	8 (11.3)	1 (8.3)	0.762
Stenosis, *n* (%)	11 (15.5)	2 (16.7)	0.918
Capillary loops thrombus, *n* (%)	14 (19.7)	3 (25.0)	0.675
TMA (%)	2 (14.3)	2 (66.7)	0.053
Renal interstitial arteriole wall thickened, *n* (%)	8 (11.3)	2 (16.7)	0.595
**AI**	11 [7, 14]	14 [10, 17]	0.159
**CI**	0 [0, 1]	0 [0, 2]	0.335

AI, activity index; CI, chronic index.

As described in **Supplementary Table S6**, there were no significant differences in the use of steroids and immunosuppressants between the two groups. However, non-compliant patients were less likely to achieve LDAS during follow-up (*P* < 0.001).

The multivariate logistic regression model showed that endothelial cell proliferation (OR 6.63, 95%CI: 1.38–31.79, *P* = 0.02) may be the risk factor for LDAS attainment (**Table 3**).

**Table 3:** Logistic regression analysis of risk factors of LDAS attainment of 272 SLE children.

Influencing factors	Univariate logistic analysis				Multi-factor logistic analysis			
	*B* value	*P* value	OR	95%CI	*B* value	*P* value	OR	95%CI
Age	0.06	0.36	0.94	0.82–1.07				
Male	0.75	0.06	2.12	0.97–4.62	1.36	0.08	3.89	0.86–17.54
Hb	0.02	0.05	1.02	1.0–1.03	0.03	0.15	1.0	0.99–1.06
Urine RBC	0.01	0.39	1.0	1.0–1.0				
Urine WBC	0.01	0.09	1.01	1.0–1.02	0.01	0.354	1.01	0.99–1.04
Mucosal ulcer	1.05	0.06	2.84	0.97–8.33	0.58	0.99	1.68	0.89–3.14
Cardiac effusion	-0.93	0.01	0.40	0.19–0.82	-0.61	0.46	0.54	0.11–2.76
Liver function damage	0.46	0.19	1.59	0.80–3.16				
Endothelial cell proliferation	1.74	<0.01	5.70	1.57–20.66	1.89	0.02	6.63	1.38–31.79

Statistically significant in univariate analysis when P <0.1 and in multivariate analysis when P <0.05.

OR, odds ratio.

### 3.3 Biological agent therapy

We also assessed organ damage and disease outcomes at baseline and the end of follow-up for patients with or without biologics (**Supplementary Table S7**). In our study, a total of 28 children were treated with biologics. The number and percentage of children who did not achieve clinical remission or LDAS after treatment with the three biologics were telitacicept (1/2, 50%), belimumab (3/4, 75%), and rituximab (7/22, 31.8%). Children with biologics achieved LDAS at a higher rate than children without biologics (*P* = 0.038) at follow-up endpoint. Organ remission was more significant in the non-biologics group (*P* = 0.037) as assessed by the pSDI <1 after the patients had been treated as efficacy.

### 3.4 Outcomes at the end of follow-up in LDAS group

**Figure 2:**
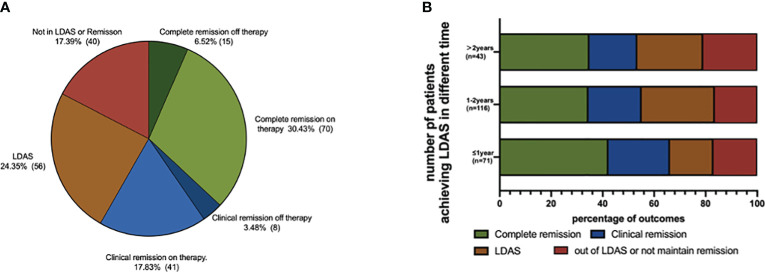
Outcomes of LDAS group pSLE at the end of follow-up. **(A)** Outcome distribution of LDAS group; 134 patients (58.26%) in LDAS group achieved remission after the medium follow-up of 36.8 (IQR, 27.0–52.9) months: complete remission off therapy (15, 6.55%), complete remission on therapy (70, 30.44%), clinical remission off therapy (8, 3.49%), and clinical remission on therapy (41, 17.90%). **(B)** Outcome distribution of reaching LDAS at different times; there were 56 patients (24.02%) still in LDAS, and the remaining 40 patients were not in any level of remission or LDAS. There was no statistical difference in the distribution of final outcomes for children who reached LDAS at different times.

Based on the T2T strategy, 134 patients (58.3%) in the LDAS group achieved remission after the medium follow-up of 36.8 (27.0–52.9) months: complete remission off therapy (15, 6.6%), complete remission on therapy (70, 30.4%), clinical remission off therapy (8, 3.5%), and clinical remission on therapy (41, 17.9%) (**Figure 2A**). There were 56 patients (24.0%) still in LDAS, and the remaining 40 patients were not in any level of remission or LDAS. There was no statistical difference in the distribution of final outcomes for children who reached LDAS at different times (**Figure 2B**).

**Figure 3:**
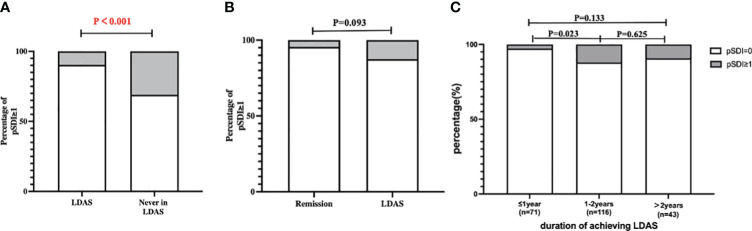
Organ damage in SLE children at the end of follow-up. **(A)** SDI of LDAS group and never in LDAS group; damage accrual assessed by pSDI ≥1 was documented in 20 (20/230, 9.62%) patients of the achieved LDAS group, which was significantly less than that of the never achieved LDAS group (13/42, 30.95%, *P* < 0.001) at the end of follow-up. **(B)** SDI of the remission group and the LDAS group; we found that the damage accrual was similar in LDAS (7/56, 12.50%) and all levels of remission (6/134. 4.48%, *P* = 0.093) according to T2T strategy at the end of follow-up. **(C)** SDI of reaching LDAS at different times; the organ damage of patients who achieved LDAS within 1 year (2/71, 2.82%), 1 to 2 years (14/116, 12.07%), and over 2 years (4/43, 9.30%) was documented by pSDI ≥1. The organ damage of patients attaining LDAS within 1 year accrued significantly less than that in 1 to 2 years (*P* = 0.028). The *P* value of the significant difference is highlighted in red.

Damage accrual assessed by pSDI ≥1 was documented in 20 (20/230, 9.6%) patients of the achieved LDAS group, which was significantly less than that of never achieved LDAS group (13/42, 31.0%, *P* < 0.001) at the end of follow-up (**Figure 3A**). However, in the 230 patients of the achieved LDAS group, we found that the damage accrual was similar in LDAS (7/56, 12.5%) and all levels of remission (6/134. 4.5%, *P* = 0.093) according to T2T strategy at the end of follow-up (**Figure 3B**).

In **Figure 3C**, the organ damage of patients who achieved LDAS within 1 year (2/71, 2.8%), 1 to 2 years (14/116, 12.1%), and over 2 years (4/43, 9.3%) was documented by pSDI ≥1. The organ damage of patients attaining LDAS within 1 year accrued significantly less than that in 1 to 2 years (*P* = 0.028). We counted outcomes at the follow-up endpoint for children who took different times to achieve LDAS, categorized as complete remission, clinical remission, LDAS, and out of LDAS or not maintaining remission. The outcome proportions of patients attaining the LDAS within 1 year, 1 to 2 years, and over 2 years showed no difference.

## 4 Discussion

This study retrospectively studied the clinical data of children with SLE from two tertiary hospitals, including clinical manifestations, laboratory data at baseline, pathological features, treatment, and clinical outcomes at the follow-up endpoint.

At baseline, the most common clinical manifestation in this study is hematologic involvement; the most common pathological type is class IV LN. The results of randomized controlled trials in adult SLE patients have shown that the use of biologics can promote remission of the disease (25). From the comparative results in our study, although the advantage of using biologics was not demonstrated from the point of view of organ damage remission, it appears that the use of biologics may promote the easier achievement of LDAS in patients. Further clinical trials are needed to assess the efficacy of using biologics in children to explore when and under what circumstances to consider using biologics.

Since the T2T strategy has been put forward to reduce organ damage and improve patients’ life quality in the treatment of some chronic diseases, a series of studies have observed that achieving LDAS can reduce severe flare and new damage during the treatment of pSLE (14, 26). In this study, we divided the included childhood lupus population into achieved LDAS group and never achieved LDAS group and analyzed their details at baseline, duration of achieving LDAS, and subsequent outcomes. We explored the risk factors for never reaching LDAS in patients with pSLE. Finally, organ damage was also used as an evaluation criterion to assess whether LDAS replaced clinical remission as a new treatment goal.

There were 84.6% of patients in our study who achieved LDAS at least once, and the median time to achieve LDAS is 15.7 months, which is similar to other studies in both adults (27) and pediatrics (28). However, only 30.9% of patients achieved LDAS within 1 year, which was less than Thailand’s report of 47% of patients achieving LLDAS within 12 months. Piga et al. found that failure to achieve LDAS within 6 months after diagnosis with SLE is often associated with early renal damage (29). In addition, the study from Cunha et al. (30) demonstrates that anemia is one of the main contributors to mortality and mortality in SLE. In our study, hematologic involvement is the most common clinical manifestation, followed by renal involvement, and the incidence of hematologic and renal involvement was relatively higher than in other centers (31). Thus, we think apart from racial differences. The later attainment of LDAS by the patients in this study may be associated with clinical characteristics at baseline levels.

In our study, a total of 42 children did not achieve LDAS, of whom 24 had SLEDAI scores >4 and 18 had SLEDAI scores <4, but the daily prednisolone dose was ≧7.5 mg. This suggests that the use of immunosuppressants should be considered for children with significant disease activity. For children whose disease activity has been controlled, early hormone reduction can be considered to avoid the negative effects caused by long-term hormone use.

In the present study, statistically significant differences were found between the two groups in terms of gender, mucosal ulcer, cardiac effusion, liver function damage, anemia, urine RBC, urine WBC, and endothelial cell proliferation at baseline. By multivariate analysis, we found that endothelial cell proliferation (*P* = 0.002) was the significant negative predictor of LDAS attainment. The renal pathology findings suggested that endothelial cell proliferation was the significant negative predictor for never reaching LDAS in pSLE patients, which was in concordance with a study proving that the more significant the manifestation of endocapillary hypercellularity in lupus nephritis, the worse the clinical prognosis (32). In this group of children, there may be a greater emphasis on immunosuppression during treatment. This is because a related adult study noted that several gene abundances in glomerular mRNA, particularly SPP1 (secreted phosphoprotein 1, also known as osteopontin), are positively correlated with endothelial cell proliferation in adult patients with LN (33). SPP1 is a pro-inflammatory molecule that plays a crucial role in autoimmune diseases by regulating Th17 cells (34). It also activates macrophages, leading to the production of growth factors directly related to cell proliferation in LN (35). In pediatric LN patients, it has also been confirmed in relevant clinical studies that children with more endothelial cell proliferation have a higher rate of clinical remission with immunosuppression (32).

Our study found that non-compliance during follow-up was also an independent risk factor for reaching LDAS (*P* < 0.001). In the course of follow-up communication with children with SLE and their parents, we found that some parents were disturbed by unscientific information from the outside world and did not have a good understanding of the disease, so they adjusted the dose of medication, stopped taking medication, and changed medication on their own, which led to recurrent disease activity. Some of the children were resistant to treatment during adolescence and did not follow medical advice, resulting in recurrent disease activity. In the management of pSLE, we usually ignore the child’s health-related quality of life (HRQoL). Donnelly et al. (36) demonstrated in the pSLE study that emotional symptoms such as anxiety and depression can be used as a predictor of HRQoL decline in children with pSLE. Therefore, in the clinical treatment work, the emotional problems of children also need to attract our attention. In addition to regular health education for parents of children, we also need to pay attention to children’s mental health problems, timely detection of psychological disorders in children, and appropriate psychological counseling to improve compliance with treatment.

In addition, this bicentric study evaluated the value of LDAS in the long-term organ damage of SLE and demonstrated that damage accrued in children attaining LDAS is significantly lower than those who never achieve LDAS. Based on the criteria of the T2T strategy, 49.3% patients of the achieved LDAS group had entered various levels of remission, a proportion a little higher than in adults (37, 38) and other studies related to pSLE (28). Furthermore, the organ damage at the end of follow-up did not present a significant difference between those patients in remission and the LDAS group, suggesting that LDAS can replace remission as a target of pSLE treatment in children. Due to the development and low-immunity characteristics of children, LDAS can be a better choice for pSLE patients who have difficulty achieving remission but simultaneously face the side effects caused by the long-term use of prednisone and immunosuppressants.

The limitations of this paper are as follows: (1) retrospective study, (2) follow-up only up to age 18, (3) small sample size despite being a bicentric study, (4) short study follow-up, and (5) limited ethnic/racial diversity.

## 5 Conclusions

In summary, we conducted a retrospective study of children with SLE from China bicentric. The clinical characteristics of SLE children at diagnosis and at the end of follow-up were evaluated. For the first time, we found that baseline endothelial cell proliferation in renal biopsy and non-compliance during follow-up were risk factors for never achieving LDAS. The use of biological agents may be beneficial to promote the realization of LDAS in patients. At the end of follow-up, the organ damage in the different remission groups was similar to that in the LDAS group, indicating that LDAS can be used as a target for pSLE treatment.

## Data availability statement

The original contributions presented in the study are included in the article/**Supplementary Material**. Further inquiries can be directed to the corresponding authors.

## Ethics statement

The studies involving humans were approved by Chongqing Children’s Hospital and Nanjing Children’s Hospital (Number: 2017[1284]). The studies were conducted in accordance with the local legislation and institutional requirements. This study was registered at the Chinese Clinical Trial Registry (ChiCTR2100046357) and the National Center for Biotechnology Information (#NCT04942314). Written informed consent for participation in this study was provided by the participants' legal guardians/next of kin.

## Author contributions

XY: Data curation, Formal analysis, Software, Writing – original draft, Writing – review & editing. JD: Data curation, Software, Writing – original draft, Writing – review & editing. QC: Data curation, Writing – original draft, Writing – review & editing. SQ: Methodology, Validation, Writing – review & editing. CJ: Data curation, Writing – review & editing. YW: Data curation, Writing – review & editing. QY: Data curation, Writing – review & editing. GZ: Data curation, Writing – review & editing. HY: Methodology, Writing – review & editing. FZ: Data curation, Writing – review & editing. QL: Supervision, Writing – review & editing. AZ: Funding acquisition, Resources, Writing – review & editing. MW: Funding acquisition, Resources, Writing – review & editing.
