# Supplementary material for: Long-term outcomes and predictive factors of achieving low disease activity status in childhood systemic lupus erythematosus: a Chinese bicentric retrospective registered study
Source: Front Immunol. 2024 Jun 10;15:1369969. doi: 10.3389/fimmu.2024.1369969 (PMC11194373; doi:10.3389/fimmu.2024.1369969)
Supplement: Supplementary file 1 [file DataSheet_1.docx]

Supplementary Material

***Long-term outcomes and predictive factors of achieving low disease activity status in childhood systemic lupus erythematosus: a Chinese bicentric retrospective registered study***

**Xixi Yu^1+^,Jia Deng^2+^, Qiuxia Chen^3+^, Shiyuan Qiu^1^, Chaohui Jiang^3^, Yiqian Wu^3^, Qin Yang^1^, Gaofu Zhang^1^, Haiping Yang^1^, Fei Zhao^3^, Qiu Li^1^, Aihua Zhang^3*^, Mo Wang^1^***

*Corresponding Author: Prof Mo Wang, Prof Aihua Zhang

Prof Mo Wang

E-mail: [wangmo_cqmu@163.com](mailto:wangmo_cqmu@163.com)

Prof Ai-Hua Zhang

Email: [zhaihua@njmu.edu.cn](mailto:zhaihua@njmu.edu.cn)

**Supplementary table 1** Definitions of clinical manifestations and renal outcomes of SLE

| **Item** | **Definition** |  |
| --- | --- | --- |
| Manifestation | |  |
| Fever | ≥38.3°C (except for fever caused by infection) |  |
| Hypertension | the systolic blood pressure and (or) diastolic blood pressure are greater than or equal to the 95th percentile of the blood pressure of children of the same sex, age and height. |  |
| Anemia | peripheral blood hemoglobin<110g/L |  |
| Leukopenia | peripheral blood white blood cell count<4×10^9^/L |  |
| Thrombocytopenia | peripheral blood platelet count<100×10^9^/L |  |
| Epilepsy | Manifestations of epileptic seizures or EEG suggestive of epileptiform discharges |  |
| Proteinuria | 24-hour urine protein quantification (24hUP) >0.5g/24h | |
| Renal function damage | eGFR<90ml/min/1.73m^2^ | |
| Hypoalbuminemia | serum albumin<25g/L | |
| Liver function damage | doubling of alanine aminotransferase and (or) aspartate aminotransferase compared with the reference value |  |
| Lupus pulmonary injury | all lung lesions that occur in patients with SLE, including those directly caused by SLE, including respiratory symptoms and effects, as well as pulmonary infections caused by various pathogens and direct damage to the lungs from therapeutic agents during treatment. |  |
| Pleurisy | Radiologically supported pleurisy |  |
| Cardiac effusion | Evaluated by cardiac color ultrasound. It can be divided into five levels: 1. Trace( less than 50ml), in the atrioventricular groove, about 3mm wide. 2. Small amount( 50-100ml), atrioventricular groove and posterior wall of left ventricle, 5mm in wider part. 3, medium, left ventricular posterior wall, apical area and right ventricular anterior wall, wider 5-10mm. 4, a large number of 300-1000ml, wrap around the whole heart, wider 10-20mm. 5. Extremely large amount, 1000-4000ml, left ventricular posterior wall 20-60mm, right ventricular anterior wall 20-40mm, visible heart swing |  |
| Treatment non-compliance | failure to follow the doctor's prescription or stop the medication during treatment, or fail to be hospitalized for regular treatment according to the plan |  |

*eGFR*, estimated glomerular filtration rate. These children were divided into two groups by weather reaching LDAS during follow-up.

**Supplementary table 2** Definition of proposed targets (T2T) in SLE

|  | Complete remission off therapy | Complete remission on therapy | Clinical remission off therapy | Clinical remission on therapy | LDAS |
| --- | --- | --- | --- | --- | --- |
| Clinical activity | No | No | No | No | Yes, but global SLEDAI≤4 |
| Serological activity | No | No | Yes | Yes | Yes, but global SLEDAI≤4 |
| Prednisone | No | ≤5mg/day | No | ≤5mg/day | ≤7.5mg/day |
| Immunosuppressive drugs | No | Yes | No | Yes | Yes |
| Antimalarials | Yes | Yes | Yes | Yes | Yes |

*LDAS*, Low disease activity status

Remission included complete remission off therapy, complete remission on therapy, clinical remission off therapy and clinical remission on therapy

**Supplementary table 3** Disease activity assessed by SLEDAI-2K at diagnosis in the achieved or never achieved LDAS group.

For disease activity, the SLEDAI-2K score was used as the basis for four groups: 0-4 for mild activity, 5-9 for moderate activity, 10-14 for high activity, and 15 or more for very high activity. We compared the distribution of SLEDAI-2K scores at the baseline level between children who achieved LDAS and never achieved LDAS. There was no significant difference in disease activity between the two groups of patients at the baseline level.

| SLEDAI-2K | Patients achieved LDAS (230) | Patients never achieve LDAS (42) | Z/χ2 | *P* |
| --- | --- | --- | --- | --- |
| Mild activity (0-4) | 12 | 0 | 2.284 | 0.131 |
| Moderate activity (5-9) | 48 | 13 | 2.075 | 0.150 |
| High activity (10-14) | 59 | 11 | 0.005 | 0.952 |
| Very high activity (≧15) | 111 | 18 | 0.416 | 0.519 |

**Supplementary table 4. Extractable nuclear antigen at baseline**

|  | **Overall**  **(n=272)** | **Patients achieved LDAS (n=230)** | **Patients never achieve LDAS (n=42)** | ***P*** |
| --- | --- | --- | --- | --- |
| ANA, n(%) |  |  |  |  |
| Negative | 29(10.7) | 25(10.9) | 4(9.5) | 0.795 |
| Positive | 243(89.3) | 205(89.1) | 38(9.1) |  |
| Anti-dsDNA , n(%) |  |  |  |  |
| Negative | 79(29.0) | 66(28.7) | 13(31.0) | 0.767 |
| ± | 19(7.0) | 19(8.3) | 0(0.0) | 0.053 |
| ＋ | 158(58.1) | 130(56.5) | 28(66.7) | 0.220 |
| ＋＋ | 16(5.9) | 15(6.5) | 1(2.4) | 0.294 |
| Total |  |  |  | 0.158 |
| Anti-ssDNA (+), n(%) | 147(54.0) | 122(53.0) | 25(59.5) | 0.438 |
| Anti-SSA (+), n(%) | 115(42.3) | 97(42.2) | 18(42.9) | 0.934 |
| Anti-SSB (+), n(%) | 35(12.9) | 29(12.6) | 6(14.3) | 0.765 |
| Anti-Sm (+), n(%) | 84(30.9) | 66(28.7) | 18(42.9) | 0.068 |
| AnuA (+), n(%) | 173(63.6) | 146(63.5) | 27(64.3) | 0.920 |
| ARPA (+), n(%) | 136(50.0) | 119(51.7) | 17(40.5) | 0.179 |
| p-ANCA, n(%) | 35(12.9) | 27(11.7) | 8(19.1) | 0.193 |
| c-ANCA, n(%) | 4(1.5) | 3(1.3) | 1(2.4) | 0.491 |
| ACA, n(%) | 43(15.8) | 35(15.2) | 8(19.1) | 0.532 |

ANA, antinuclear antibodies; anti-dsDNA, anti-double-stranded DNA; anti-ssDNA, anti-single-stranded DNA; AnuA, anti-nucleosome antibody; anti-P, Anti-ribosomal-P antibodies; SSA, Sjögren's syndrome A; SSB, Sjögren's syndrome B; ARPA:Anti-Ribosomal P Protein Antibody; ACA, anti-cardiolipin antibody; ANCA, anti-neutrophil cytoplasm antibodies.

**Supplementary figure 1** LN pathological types in 87 patients who underwent renal biopsy. **a** total. **b** LDAS and never in LDAS.

Renal biopsy was done in 87 patients (*Supplementary Figure S1a*). Class IV LN was the most common type(35 cases, 42.17%). There were 15 cases (18.07%) of class IV+V LN, 11 cases (13.25%) of type V LN, and 6 cases (7.23%) of type II LN. Type III+V LN was the least type in these patients. No class I and class VI LN were found in this study. The median acute index (AI) is 11.5 (IQR, 7.0-14.0) and the median chronic index (CI) is 0 (IQR, 0-1.0). In the distribution of renal pathology types, we did not find a statistical difference between the two groups(*Supplementary Figure S1b*). 75 (86.21%) of 87 renal biopsy children have achieved LDAS. Class IV LN was the most common type in both achieved LDAS (30 cases, 40.23%) and never achieved LDAS (5 cases, 41.67%).


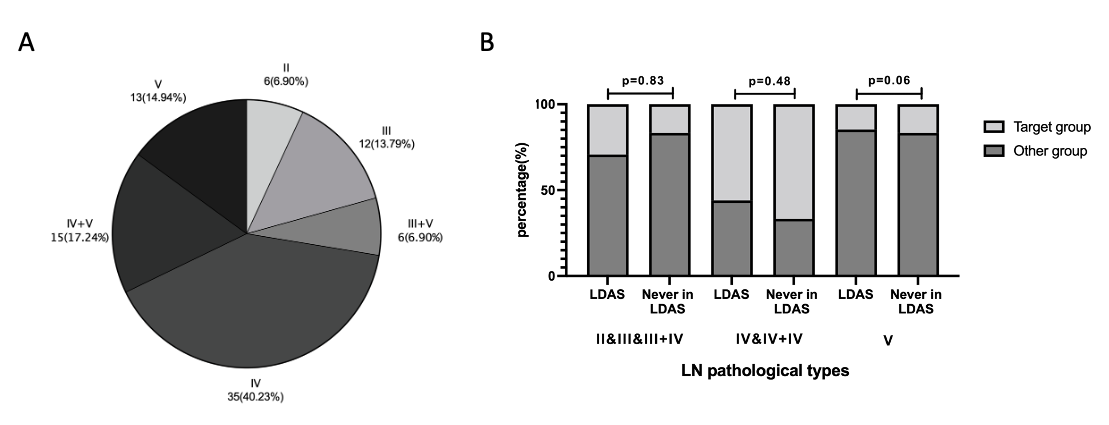


**Supplementary table 5** The difference between females and males in clinical manifestation and laboratory characteristics at baseline.

|  | Female | Male | Z/χ2 | *P* |
| --- | --- | --- | --- | --- |
| **Clinical manifestation** |  |  |  |  |
| Fever, n(%) | 129(56.6) | 19(43.2) | 2.669 | 0.102 |
| Hypertension, n(%) | 44(19.3) | 8(18.2) | 0.030 | 0.863 |
| Rash, n(%) | 154(67.5) | 30(68.2) | 0.007 | 0.934 |
| Mucosal ulcer, n(%) | 47(20.6) | 10(22.7) | 0.099 | 0.753 |
| Alopecia, n(%) | 24(10.5) | 2(4.6) | 1.526 | 0.274 |
| Anemia, n(%) | 175(76.8) | 30(68.2) | 1.460 | 0.227 |
| Leucopenia, n(%) | 109(47.8) | 19(43.2) | 0.317 | 0.574 |
| Epilepsy, n(%) | 64(28.1) | 11(25) | 0.174 | 0.677 |
| Neurological involvement, n(%) | 24(10.5) | 1(2.3) | 2.103 | 0.147 |
| Proteinuria, n(%) | 134(58.8) | 26(59.1) | 0.002 | 0.969 |
| Hematuria, n(%) | 103(45.2) | 16(36.4) | 1.164 | 0.281 |
| Pyuria, n(%) | 50(21.9) | 10(22.7) | 0.014 | 0.907 |
| Renal function damage, n(%) | 43(18.9) | 6(13.6) | 0.701 | 0.403 |
| Lupus pulmonary injury, n(%) | 90(39.5) | 17(38.6) | 0.011 | 0.917 |
| Pulmonary hypertension, n(%) | 5(2.2) | 1(2.3) | 0.001 | 1.000 |
| Pleuritis or pleural effusion, n(%) | 81(35.5) | 11(25) | 1.826 | 0.177 |
| Cardiac effusion, n(%) | 49(21.5) | 3(6.8) | 5.135 | 0.023 |
| Arthritis, n(%) | 82(36) | 15(34.1) | 0.056 | 0.812 |
| SLEDAI-2K | 14.0 [9.0,20.8] | 14.0 [9.0,18.8] | -0.501 | 0.617 |
| pSDI | 72(31.6) | 15(34.1) | 0.107 | 0.744 |
| **Laboratory characteristics** |  |  |  |  |
| WBC , ×10^9^/L | 4.1[3.0,5.8] | 4.2[2.9,5.9] | -0.227 | 0.820 |
| PLT , ×10^9^/L | 149.0[96.5,231.8] | 163.0[104.8,248.3] | -0.423 | 0.672 |
| Hb , g/L | 94.0[82.0,108.0] | 103.0[88.0,113.0] | -1.763 | 0.078 |
| ESR, mm/h | 44.0[23.0,72.0] | 31.0[18.0,54.0] | -1.914 | 0.056 |
| Urine RBC, /ul | 14.0[1.6,121.0] | 9.5[1.3,141.3] | -0.432 | 0.637 |
| Urine WBC, /ul | 10.5[3.8,29.7] | 9.0[1.1,26.3] | -0.765 | 0.444 |
| Urine pathological cast, n(%) | 72.0[31.0,58.0] | 12.0[27.0,27.0] | -0.225 | 0.829 |
| 24h-UP, g/day | 0.6[0.2,1.8] | 0.7[0.3,2.3] | -0.539 | 0.590 |
| Alb, g/L | 33.9±7.8 | 33.3±9.0 | -0.492 | 0.623 |
| ALT, U/L | 30.0[15.6,56.2] | 32.9[19.1,57.4] | -0.753 | 0.451 |
| AST, U/L | 33.3[24.1,60.0] | 39.5[25.5,64.3] | -0.896 | 0.370 |
| BUN, mmol/L | 4.7[3.6,8.5] | 6.0[3.8,7.7] | -1.062 | 0.288 |
| Scr, mmol/L | 47.0[36.0,70.1] | 43.4[38,60.5] | -0.495 | 0.621 |
| eGFR , ml/min/1.73m^2^ | 147.3[101.4,189.5] | 158.3[139.3,193.8] | -1.657 | 0.097 |
| Coombs test positivity, n(%) | 177.0(77.6) | 30.0(68.2) | 1.811 | 0.178 |
| C3 , g/L | 0.4[0.2,0.5] | 0.3[0.2,0.5] | -0.796 | 0.359 |
| C4, g/L | 0.1[0.0,0.1] | 0.1[0.0,0.1] | -0.076 | 0.939 |
| Autoantibody positivity |  |  |  |  |
| ANA, n(%) | 203(89.0) | 40(90.9) | 0.136 | 0.712 |
| dsDNA, n(%) | 177(77.6) | 33(75.0) | 0.145 | 0.703 |
| ssDNA, n,(%) | 120(52.6) | 27(61.4) | 1.132 | 0.287 |
| SSA, n(%) | 98(43.0) | 17(38.6) | 0.285 | 0.593 |
| SSB, n(%) | 30(13.2) | 5(11.4) | 0.106 | 0.745 |
| Sm, n(%) | 63(27.6) | 21(47.7) | 6.978 | 0.008 |
| AnuA, n(%) | 146(64.0) | 27(61.4) | 0.114 | 0.736 |
| ARPA, n(%) | 107(46.9) | 29(65.9) | 5.314 | 0.021 |
| p-ANCA, n(%) | 32(14.0) | 3(6.8) | 1.713 | 0.191 |
| c-ANCA, n(%) | 3(1.3) | 1(2.3) | 0.233 | 0.508 |
| ACA, n(%) | 36(15.8) | 7(15.9) | 0.000 | 0.984 |

**Supplementary table 6** Therapy of patients achieved or never achieved LDAS at baseline.

Treatment is divided into induction remission therapy and maintenance remission therapy. For mildly active pSLE, hydroxychloroquine or NSAIDs are used to treat skin and joint symptoms in the induction remission phase, when treatment is not effective, a low dose of steroids, prednisone <0.5 mg/(kg-d) or <5 mg/d clouds be considered, For moderately active pSLE, steroids dose 0.5-1.0mg/(kg-d), combined with immunosuppressive agents or biologics if steroids control is poor or difficult to reduce. For severely active pSLE, use prednisone ≥1.0 mg/(kg-d) or equivalent doses of other steroids in combination with immunosuppressive agents. Immunosuppressive agents are usually chosen from: 1) Cyclophosphamide (CTX): 8-12 mg/kg/day for 2 days every 2 weeks, with extended dosing intervals after 6 courses for 1-3 years. 2) Mycophenolate mofetil (MMF): 30-40 mg/kg/day divided into 2 oral doses, with the total daily dose not exceeding 2g. 3) Tacrolimus (Tac): 0.1-0.15 mg/kg/day, with the maximum daily dose not exceeding 4 mg, with monitoring of the effective blood concentration maintained at 5-15 μg/L. 4) Methotrexate (MTX): 10-15 mg/ m^2^ once a week and 5mg of oral folic acid on the second day. Biologic options: 1) Belimumab: 10mg/kg intravenously for the first 3 doses every 2 weeks, then every 4 weeks. 2) Rituximab: 375 mg/m2 weekly for 2-4 weeks.

|  | Patients achieved LDAS (230) | Patients never achieve LDAS (42) | Z/χ2 | P value |
| --- | --- | --- | --- | --- |
| Hydroxychloroquine, n(%) | 188(81.7) | 31(73.8) | 1.423 | 0.233 |
| High-dose methylprednisolone, n(%) | 179(77.8) | 34(81.0) | 0.204 | 0.651 |
| Immunoglobulin, n(%) | 18(7.8) | 7(16.7) | 2.351 | 0.125 |
| Prednisone dose, mg/d | 52.5[45.0, 60.0] | 52.5[45.0,60.0] | -0.793 | 0.428 |
| Prednisone, n(%)] | 68(29.6) | 11(26.2) | 0.196 | 0.658 |
| Prednisone +CYC, n(%) | 84(36.5) | 12(28.6) | 0.983 | 0.321 |
| Prednisone +MMF, n(%) | 50(21.7) | 14(33.3) | 2.653 | 0.103 |
| Prednisone +CYC+MMF, n(%) | 25(10.9) | 5(11.9) | 0.039 | 0.792 |
| Biologics, n(%) | 21(9.1) | 7(16.7) | 2.184 | 0.165 |
| Treatment non-compliance, n(%) | 9(3.9) | 11(26.2) | 22.706 | ＜0.001 |

Medication route of High-dose methylprednisolone and immunoglobulin were intravenous infusion. Immunoglobulin> 1-2g/kg. Biologics include rituximab and belimumab. CYC: Cyclophosphamide; MMF: mycophenolate mofetil.

**Supplementary table 7** Characteristics of patients with or without using biologics at baseline and the end of follow-up.

|  | Patients with using biologics(28) | Patients without using biologics(244) | Z/χ2 | P value |
| --- | --- | --- | --- | --- |
| Baseline |  |  |  |  |
| SLEDAI-2K | 15.50 [13.0, 21.0] | 14.00 [9.0, 20.0] | -1.663 | 0.096 |
| pSDI≥1, n(%) | 12(42.9) | 75(30.7) | 1.696 | 0.193 |
| Therapy |  |  |  |  |
| Prednisone +CYC, n(%) | 11(39.3) | 85(34.8) | 0.218 | 0.641 |
| Prednisone +MMF, n(%) | 18(64.3) | 54(22.1) | 22.932 | <0.001^*^ |
| Prednisone +CYC+MMF, n(%) | 2(7.1) | 28(11.5) | 0.480 | 0.751 |
| Follow-up |  |  |  |  |
| Achieved LDAS | 21(75.0) | 209(85.7) | 2.184 | 0.165 |
| Outcome at endpoint |  |  |  |  |
| Remission, n(%) | 8(38.1) | 126(60.3) | 3.865 | 0.049 |
| LDAS, n(%) | 9(42.9) | 47(22.5) | 4.298 | 0.038 |
| Not in remission or LDAS, n(%) | 4(10.1) | 36(17.2) | 0.044 | 0.834 |
| pSDI≥1, n(%) | 7(33.3) | 21(10.1) | 9.677 | 0.002 |
| Damage mitigation, n(%) | 5(41.7) | 54(72.0) | 4.361 | 0.037 |
